# Public Street Lights Increase House Infestation by the Chagas Disease Vector *Triatoma dimidiata*

**DOI:** 10.1371/journal.pone.0036207

**Published:** 2012-04-27

**Authors:** Freddy Santiago Pacheco-Tucuch, Maria Jesus Ramirez-Sierra, Sébastien Gourbière, Eric Dumonteil

**Affiliations:** 1 Laboratorio de Parasitología, Centro de Investigaciones Regionales “Dr. Hideyo Noguchi," Universidad Autónoma de Yucatán, Mérida, Yucatán, Mexico; 2 UMR 5244 CNRS-UPVD ‘Ecologie et Evolution des Interactions,’ Université de Perpignan Via Domitia, Perpignan, France; 3 Centre for the Study of Evolution, University of Sussex, School of Life Sciences, University of Sussex, Brighton, United Kingdom; 4 Department of Tropical Medicine, School of Public Health and Tropical Medicine, Tulane University, New Orleans, Louisiana, United States of America; U.S. Naval Medical Research Unit Six (NAMRU-6), Peru

## Abstract

*Triatoma dimidiata* is one of the primary vectors of Chagas disease. We previously documented the spatio-temporal infestation of houses by this species in the Yucatan peninsula, Mexico, and found that non-domiciliated triatomines were specifically attracted to houses. However, the factors mediating this attraction remained unclear. Artificial light has been known for a long time to attract many insect species, and therefore may contribute to the spread of different vector-borne diseases. Also, based on the collection of different species of triatomines with light traps, several authors have suggested that light might attract triatomines to houses, but the role of artificial light in house infestation has never been clearly demonstrated and quantified. Here we performed a spatial analysis of house infestation pattern by *T. dimidiata* in relation to the distribution of artificial light sources in three different villages from the Yucatan peninsula, Mexico. In all three villages, infested houses were significantly closer to public street light sources than non-infested houses (18.0±0.6 *vs* 22.6±0.4 m), and street lights rather than domestic lights were associated with house infestation. Accordingly, houses closer to a public street lights were 1.64 times more likely to be infested than houses further away (OR, CI95% 1.23–2.18). Behavioral experiments using a dual-choice chamber further confirmed that adult male and females were attracted to white light during their nocturnal activity. Attraction was also dependent on light color and decreased with increasing wavelength. While public lighting is usually associated with increased development, these data clearly show that it also directly contributes to house infestation by non-domiciliated *T. dimidiata*.

## Introduction

Artificial light is an important factor potentially influencing insect vector dispersal [1]. Indeed, it is well known that several vectors species from the genera *Aedes*, *Culex* and *Anopheles* are attracted by artificial light [2], [3]. Nonetheless, the potential contribution of artificial light to the spreading of these vectors and of the pathogens they transmit has rarely been evaluated. Chagas disease is a major parasitic disease, caused by *Trypanosoma cruzi* and transmitted to mammalian hosts by several species of hematophagous triatomines. Several field studies have successfully used light traps for the collection of different species of sylvatic triatomines [4]–[9], although triatomines demonstrate negative phototaxis [10].

Additional studies on the behavior of *Triatoma infestans* and *Rhodnius prolixus*, two primary Chagas disease vector species, revealed a significant bias in their take-off activity toward a light source, confirming some level of attraction to light, rather than its use for menotaxis [11]. Based on these scant observations, several authors have hypothesized that triatomines can be attracted to houses by artificial lights, but epidemiological studies investigating the level of increased risk associated with artificial lighting use are limited [1]. For example, as early as in the 1930s, Campos wrote: “How to explain the increasing dispersal of *Triatoma dimidiata* in our houses? It is easy. The cause is the amplitude of the public service of electric light in the city, a service which intensification in the past years has highly favored the dissemination of the dangerous insect, attracting it with the strength of the light from the urban surroundings and favoring its entry in the inhabited perimeter" [12]. More recently, light has also been incriminated for house infestation by *Triatoma brasiliensis* and *Triatoma pseudomaculata* in northeastern Brazil [6], *Rhodnius robustus* in western Venezuela [13], *Rhodnius pallescens* in Central America [14], based mostly on the collection of these species with light traps. Therefore, studies are needed to determine the attraction of artificial light in relation to house infestation by triatomines.

*Triatoma dimidiata* is one of the primary vector species of Chagas disease, with a widespread distribution in southern Mexico, central America and parts of Venezuela, Colombia, Ecuador and Peru [15]. It is present in a variety of environments and habitats and poses particular challenges for vector control programs, since it has a well documented ability to re-infest houses following conventional insecticide spraying [16]–[20]. A better understanding of the process by which *T. dimidiata* disperses and is attracted to domestic areas would provide a better understanding of the biology of this species, ultimately leading to improved vector control [16], [21], [22]. In the Yucatan peninsula, Mexico, *T. cruzi* seropositivity in humans reaches about 1% in urban areas, and up to 5% in rural villages [23]. In this region as well as in Belize, *T. dimidiata* behaves as a non-domiciliated triatomine able to transiently infest houses on a seasonal basis, but with very limited ability to colonize houses [24]–[27]. Recent molecular studies have established that *T. dimidiata* is in fact a complex encompassing several sibling species [28]–[31]. Two of these sibling species are present in the Yucatan peninsula, as well as hybrids between them, but all behave similarly [28], [32] and cannot be distinguished easily by morphometry [33]. Importantly, analysis of *T. dimidiata* dispersal process in a rural village clearly indicated that triatomines did not disperse randomly within the village, but were particularly attracted to houses [34]. In that respect, we have previously documented that infestation follows a marked spatial structure, preferentially affecting houses that are closer to the bushes and secondary vegetation surrounding rural villages [35], and urban areas [36]. However, it is very likely that additional factors are responsible for differences in attractiveness of houses to dispersing triatomines, and artificial light may contribute to infestation as suggested above.

The purpose of the present study was thus to test whether artificial light could play a role in house infestation by *T. dimidiata* in the Yucatan peninsula, Mexico. To assess the role of artificial light in regards to infestation, we performed a detail analysis of lighting and infestation spatial patterns in several rural villages, and investigated the behavioral response of *T. dimidiata* to light in behavioral experiments.

## Materials and Methods

### Study area and field studies

The study was carried out in the rural villages of Bokoba (21.01°N, 89.07°W), Teya (21.05°N, 89.07°W) and Sudzal (20.87°N, 88.98°W), which were located about 15 to 20 km apart in the central part of the Yucatan state, Mexico. The villages are surrounded by a mixture of secondary bush vegetation and agricultural land. There are a total of 570, 702 and 416 houses in Bokoba, Teya and Sudzal, respectively, all of which were geo-referenced [35], for a population of 1973, 1924 and 1527 inhabitants. Triatomine infestation was determined by community participation as in previous studies, with inhabitants bringing the vectors found in their houses to the local health center of each village for identification [35], [37]. The study was approved by the Institutional Bioethics Committee of the Autonomous University of Yucatan.

Artificial light sources of the villages were also identified and geo-referenced. We distinguished two potential sources: public lights corresponding to light posts illuminating the streets and parks, and house/peridomestic lights corresponding to houses with an outdoor light illuminating their entrance or backyard (peridomiciles). Light intensity of both sources was measured at a distance of 4 to 5 m from the light source with a Steren HER-410 meter, between 20:00 to 23:00 hours. Indoor lighting was not investigated in this study as all houses within the villages had electrical power and similar indoor lighting, which therefore was unlikely to contribute to differences in infestation between houses.

### Spatial analysis of field data

All coordinates were entered in a GIS database and maps of infested/non-infested houses and of the position of the different light sources were produced in Qgis 1.7 [38]. We then calculated the distance from each house to the closest light source. The relationship between house infestation and light sources was evaluated by comparing the distance from infested and non-infested houses to the different light sources. The statistical significance of potential differences was assessed by empirical permutation tests based on 100,000 Monte Carlo resampling of an identical number of houses within the villages, which simulated random distributions of their distances to light sources [35], [39]. Odd ratio for infestation as a function of the proximity of light sources to houses was also calculated.

### Behavioral experiments

The attraction of *T. dimidiata* toward light sources was studied in a dual choice chamber. The chamber consisted of a rectangular arena (0.5 m×1 m×0.5 m) made with a wooden floor, plastic insect screen walls, and a glass ceiling. The arena had two outgoing dead-end tunnels of 10 cm in diameter and 50 cm long, which could be lit using light-emitting diodes (LEDs) of distinct wavelength [3]. We assessed the effect of white light (∼400 to 750 nm), as well as blue (430 nm), green (500 nm), yellow (590 nm) and red (630 nm) lights. Field-collected and first-laboratory generation insects were kept under natural photoperiod, and starved for 10–25 days prior to the experiments. Room temperature was kept at 24±2°C. Adult male and female *T. dimidiata* or 5^th^ stage nymphs (5–30 individuals per experiment) were placed in the middle of the chamber at dusk, in an artificial refuge made of folded papers, and videotaped until morning to assess their activity during the night. The number of triatomines entering the lit and the unlit tunnels, time of entry, as well as the hourly activity pattern were measured. The position of the light was switched from one tunnel to the other for each experiment. Also, control experiments were performed without light to control for potential bias of the chamber or in triatomine behavior.

Attraction to light was calculated as an attraction index defined as: AI = (T−C)/(T+C+O), where T = number of triatomines that entered the lit tunnel, C = number of triatomines that entered the dark control tunnel, and O = number of triatomines that remained in the arena outside the tunnels [40]. An attraction index of 0 thus corresponds to random visits in both tunnels without any preference, positive values indicate attraction to the lit tunnel, and negative values to the dark tunnel. Statistical significance of the attraction was determined by χ^2^ tests comparing AI in the presence of light with control AI corresponding to both tunnels in the dark.

## Results

### Spatial distribution of house infestation and light sources

**Figure 1:**
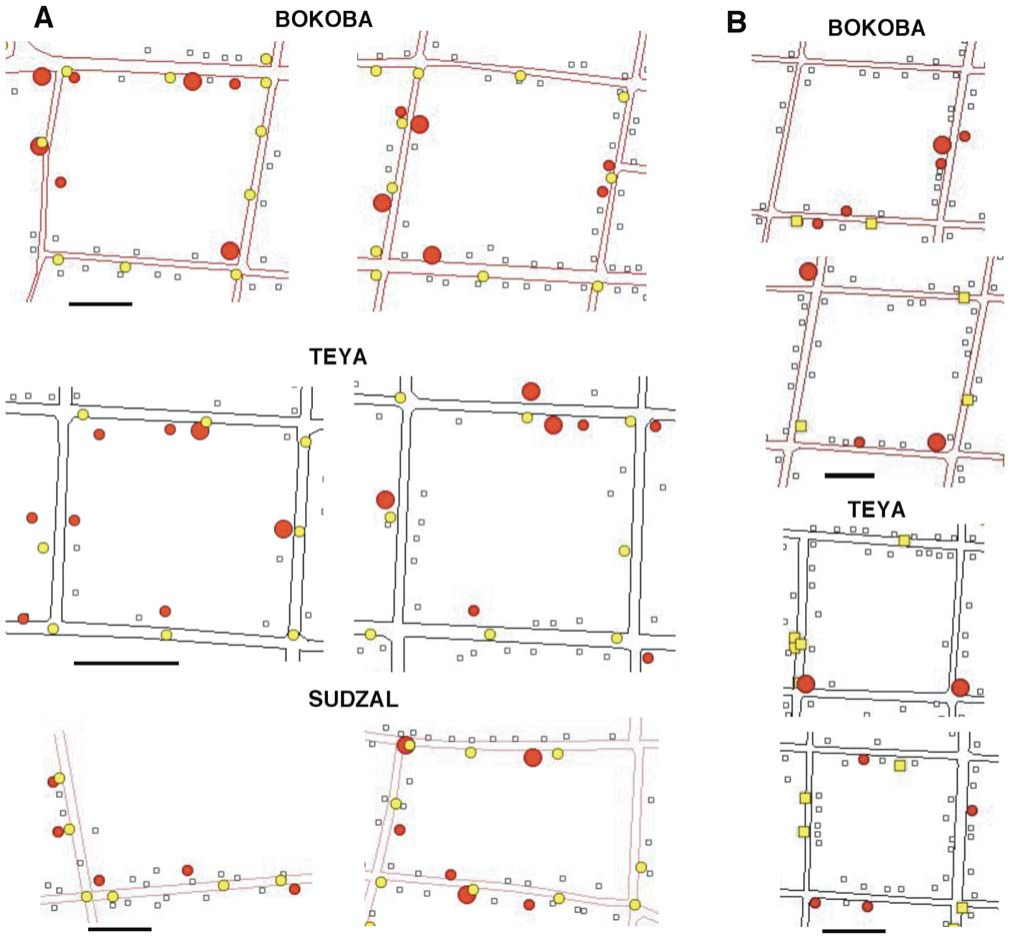
Spatial patterns of infestation and artificial lights. (A) Examples of spatial distribution of infested (red circles) and non-infested houses (empty squares) and public street lights (yellow circles) from parts of the indicated villages. (B) Examples of spatial distribution of infested and non-infested houses and peridomicile lights (yellow squares) in the indicated villages. Lines represent the streets. Scale bars: 50 m.

A total of 1,466 houses were included in the study, of which 214 (14.6%) were found to be infested by *T. dimidiata*. We also geo-referenced a total of 565 public lights (135–228 lights per village), and identified 229 houses (15.6%) with outdoor lights in their peridomicile. The relationship between infestation by *T. dimidiata* and the presence of artificial light was first assessed by mapping their respective spatial distribution. As presented in Figure 1A, we observed many occurrences of infested houses very close to public street lights, while un-infested houses were frequently further away from these lights. These results indicated that public lights may be associated with infestation. On the other hand, lights in the peridomiciles did not exhibit the same distribution. Infestation did not appear to occur preferentially in or close to houses with a light in their backyard, but rather randomly with regard to this source of light (Fig. 1B).

**Figure 2:**
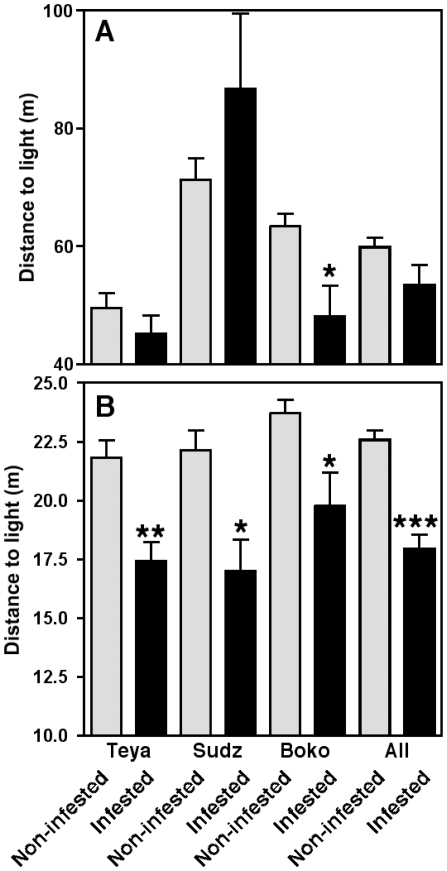
Observed distance from houses to the nearest light according to their infestation status. (A) Distance to the nearest peridomicile light of infested (black bars) and non-infested houses (light gray bars). (B) Distance to the nearest public street light of infested and non-infested houses. Data are presented as mean ± SEM. *, **, and *** indicate a significant difference between infested and non-infested houses (*P*<0.05, <0.01 and <0.001, respectively).

For a quantitative evaluation of these patterns, we compared the distance of infested and non-infested houses to the closest light sources, as measured from maps of the entire villages. Analysis of the spatial distribution of houses with outdoor lights in their peridomiciles indicated a variable and inconsistent relationship with infestation in the different villages (Fig. 2A). Indeed, infested houses were located significantly closer to an outdoor light only in the village of Bokoba (*P* = 0.028), while no significant differences were observed in the other two villages (*P* = 0.45 and 0.21, in Teya and Sudzal, respectively). Thus, for all the villages, the average distance to an outdoor light in a peridomicile was similar for infested and non-infested houses (Fig. 2A). In contrast, infested houses were significantly closer to public lights than non-infested houses in all three studied villages (Fig. 2B, *P* = 0.008, 0.044 and 0.039 for the villages of Teya, Sudzal and Bokoba, respectively). Infested houses were on average 18.0±0.6 m from a public light *vs* 22.6±0.4 m for non-infested houses (*P*<0.001). Accordingly, over 50% of infested houses were located near a public light compared to less than 40% of non-infested ones (Table 1). The proximity of a public light resulted in a 1.64 fold increase in the likelihood of a house being infested (95% confidence interval: 1.23–2.18).

**Table 1:** Relationship between infestation and the proximity of street lights.

	Close to a street light (<17 m)	Far from a street light (>17 m)
Infested houses	111 (51.9%)	103 (48.1%)
Non-infested houses	581 (39.6%)	885 (60.4%)

The proximity of a public street light resulted in a 1.64 fold increase in the likelihood of a house being infested (95% confidence interval: 1.23–2.18).

These data provided a clear field evidence that the proximity of a public light, but not of a house light in the peridomicile, can be a key factor making some houses more attractive than others to *T. dimidiata*. To assess the potential differences between the two light sources, we compared their intensity. However, no significant differences were observed between the brightness of public street lights and lights in the peridomiciles (22±1 *vs* 23±3 lux, *P* = 0.29, respectively). Thus, other characteristics are likely responsible for their different contribution to infestation and we further investigated triatomine's behavior in response to light to clarify this point.

### Behavior of *T. dimidiata* to light

**Figure 3:**
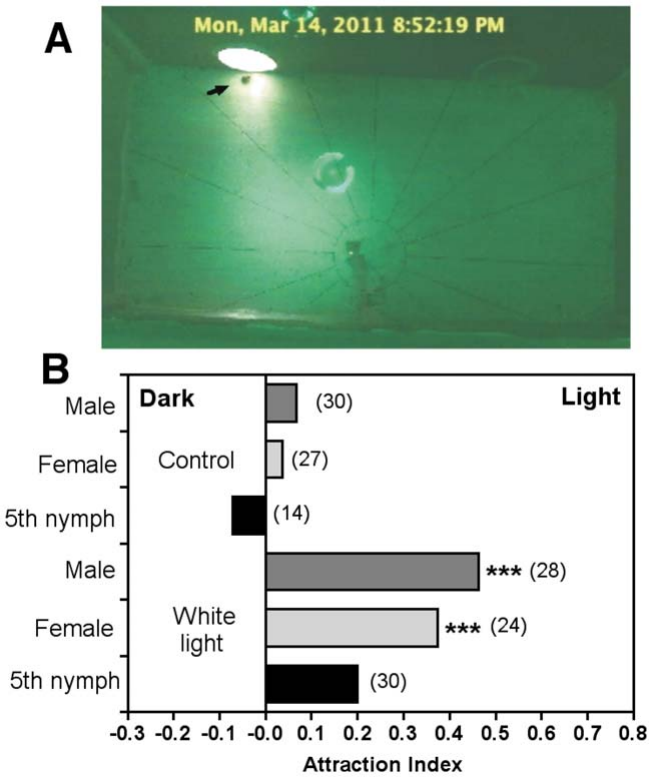
Attraction of *T. dimidiata* by white light in a dual-choice chamber. (A) Screen capture image of the chamber showing a triatomine about to enter the lit tunnel (arrow). (B) Attraction by light of adult male and female and 5^th^ nymphal stage triatomines. The attraction index was calculated as described in the methods, with positive values indicating preference for the lit tunnel, negative values for the dark tunnel, and 0 indicates random distribution in both tunnels. Control experiments were carried out with both tunnels in the dark. *** indicates a significant difference with the control without lights (*P*<0.001) and numbers in parenthesis indicate the number of triatomines tested.

**Figure 4:**
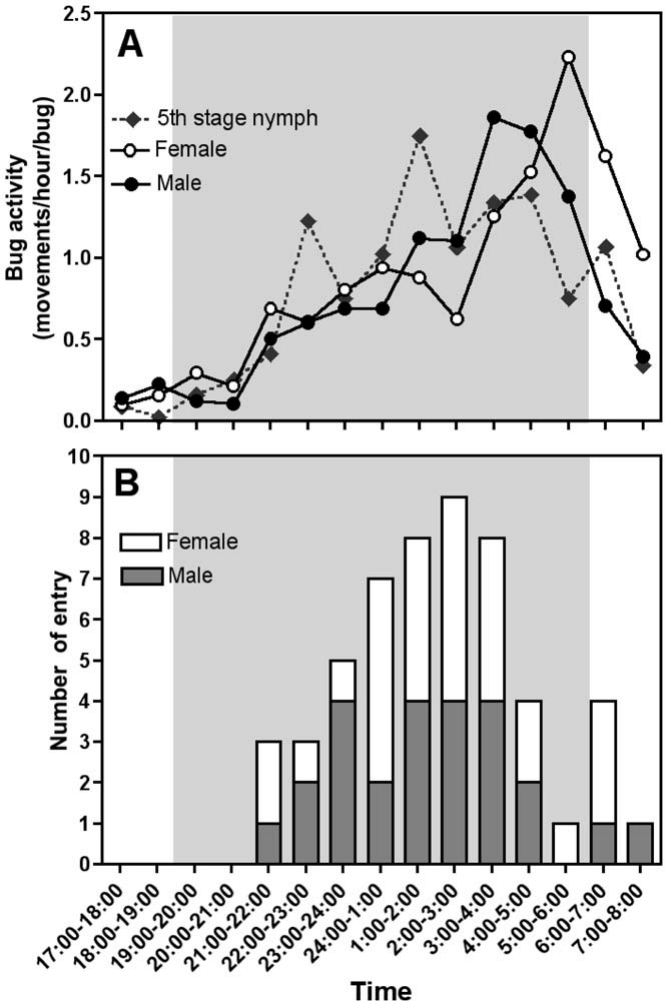
Activity pattern and light attraction of *T. dimidiata* during the night. (A) The number of individual movements per hour was determined from the analysis of the video recordings of adult males, females and 5^th^ stage nymphs. (B) The time adult males (dark gray bar) and females (white bar) entered the tunnel with light was recorded. Shaded gray areas indicate the night period.

We used a dual-choice chamber to investigate *T. dimidiata* attraction to light in darkness. Initial control experiments in the absence of light sources in the two tunnels confirmed the absence of bias in our experiments as all triatomines tested, including adult males, females and 5^th^ stage nymphs, visited both tunnels in equal proportion (Fig. 3). When given the choice of either a dark tunnel or a lit one, both adult male and female *T. dimidiata* were significantly attracted to white light, with around 40% of the triatomines entering the lit tunnel during the night (Fig. 3; *P*<0.001 for both, and Supplementary Video S1). On the other hand, attraction of 5^th^ stage nymphs was limited and did not reach statistical significance (*P* = 0.21). Analysis of activity patterns during the night indicated that the lack of attraction of 5^th^ stage nymphs was not due to inactivity in the chamber, as these presented a very similar pattern of activity during the night as adult males and females (Fig. 4A). All triatomines started to move around the chamber about 2 hours after dusk and their activity increased over the night, reaching a maximum in the early morning, and then rapidly stopped just before day breaks. We also evaluated if light attraction varied during the night, and found that triatomines entered the lit tunnel at any time during the night, between 21:00 and 8:00 hours. However, the number of entry varied according to time and the maximum was observed in the middle of the night, between 1:00 and 4:00 hours (Fig. 4B).

**Figure 5:**
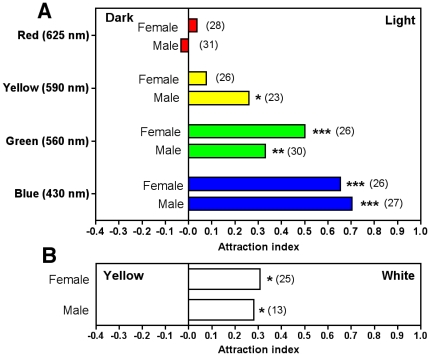
Attraction of *T. dimidiata* by light of different wavelength. (A) Light emitting diodes (LEDs) of the indicated wavelength were used in the dual choice chamber and attraction measured for adult male and female triatomines. (B) Triatomines were also given the choice between white and yellow light. *, **, and *** indicate a significant difference with the control without lights (*P*<0.05, <0.01 and <0.001, respectively) and numbers in parenthesis indicate the number of triatomines tested.

We further assessed if light attraction varied according to wavelength, and tested LEDs of different colors ranging from blue (430 nm) to red (625 nm). There was no significant difference in the response of adult males and females to the different wavelength tested; both were strongly attracted to short wavelength light (blue) and attraction gradually decreased as the wavelength increased (Fig. 5A). Thus, yellow light was barely attractive while red light was not attractive at all. Differences in attraction were not due to differences in activity patterns as triatomines presented a similar activity during the night at all wavelength tested (data not shown). We then tested attraction when triatomines were given the choice of white versus yellow lights in the chamber. In that situation, triatomines were significantly attracted by white light and not by yellow light (Fig. 5B), which could thus be used to avoid attraction.

## Discussion

Artificial light has been known for a long time to attract many insect species and light traps have been used to collect different species of triatomines, including *T. dimidiata*. Based on this, several authors have suggested that light might attract triatomines to houses, but the role of artificial light in house infestation has never been clearly demonstrated and quantified. The results of this study present the first evidence demonstrating that the presence of artificial light directly influences house infestation by *T. dimidiata* in field conditions. The data indicate that infested houses were located closer to public lights than non-infested houses, with the proximity of a public light increasing the probability of a house being infested 1.64 fold. It is remarkable that a spatial pattern associating infestation and artificial lights could be detected in all three studied villages, in spite of the many potential confounding factors present (heat, odors, climate, hosts, vegetation, etc…) [11]. It is also interesting to note the specific role of street lights, and not that of lights from the peridomicile of the houses. Differences in light intensity did not seem to explain this discrepancy. Alternatively, it is possible that the time periods the various artificial lights are used together with the activity of triatomines may explain the difference in attractiveness between light sources as most families turn off their lights before going to sleep. Therefore, these lights are likely only present for the early part of the night, typically until 22:00 to 23:00 hours. The results from our experiments suggest that *T. dimidiata* attraction to light occurred preferentially in the later half of the night (between 1:00 to 4:00 hours), which does not coincide with the presence of lights in the houses. In contrast, public street lights remain on from dusk until dawn, year round, and provide a constant source of artificial light. Further, public street lights may be much more visible over larger distances, due to their height, light intensity, and their location along streets, while peridomestic lights are typically less visible due to the peridomestic vegetation and other enclosures. We may nonetheless be underestimating the contribution of lights in the peridomiciles. Indeed, these were only observed on a single night, and their use may be highly variable from day to day, month to month, and family activities, making this light pattern more difficult to assess. A more detailed recording of light usage, and particularly during the infestation season, will provide a clearer understanding of light usage in house infestation by *T. dimidiata*.

Behavioral assays in our dual-choice chamber further confirmed that *T. dimidiata* is strongly attracted to low intensity white light, which corresponds to public/house lighting at a distance of approximately 50 m. This is in good agreement with the idea that light may mediate attraction of a house over rather large distances of typically 20 to 100 m, as determined in our previous modeling of dispersal [34]. Other attractive stimuli have been described for some triatomine species, such as host odors, CO_2_, or heat, which are believed to play a key role in finding hosts for blood feeding [41]. However, it is unlikely that these stimuli play an important role at distances comparable to that of light. Our data also confirm previous observations on *T. infestans* and *R. prolixus*, since both species orient their take-off activity toward a light source [11]. Analysis of attraction with light of different wavelength suggested some color vision in *T. dimidiata*, as attraction decreased with increasing wavelength (at a constant intensity), although more tests would be required to determined the extent of color discrimination in this species [42], [43]. It is difficult to speculate on how useful or critical it is for these nocturnal triatomines to have color vision, and it may be a relic from their diurnal ancestors, as color vision appeared conserved across many insect species in spite of major differences in lifestyles and habitats [2], [42], [44]. More detailed analysis of spectral sensitivity of the photoreceptors expressed in triatomine eyes would provide a better understanding of their visual abilities. Our observations of a limited sensitivity to longer wavelengths (>560 nm) in *T. dimidiata* appears in agreement with a previous study on the photonegative behavior of *T. infestans* [10], suggesting a similar visual sensitivity in both species. Consistent with these observations, a BLAST search of *R. prolixus* draft genome for sequences homologous to opsin photoreceptors identified a single gene encoding for a protein with high similarity to Rh6 (62% identity), Rh2 (57% identity) and Rh1 opsins (56% identity) from *Drosophila melanogaster*, which maximum sensitivity is at 508, 420 and 478 nm, respectively [45], suggesting that sensitivity to short wavelengths (<560 nm) may be conserved across the Triatominae subfamily.

Importantly, from a public health perspective, the lower sensitivity of triatomines to long wavelengths may open the way to the use of alternative light sources (yellow lights for example) of reduced attractiveness to triatomines, which may help vector control efforts. Indeed, we previously determined that a simple reduction of house attractiveness to triatomines could reduce house infestation by up to 60% [21]. Alternatively, using shorter wavelength light traps (<560 nm) may increase their efficacy for triatomine collections. Finally, our geospatial approach could be useful to assess the role of artificial light in house infestation by other triatomine species, given that most have been collected successfully with light traps [4]–[6], [8], [9], as well as other species of insect vectors [1]. Our data also suggest that public light spatial patterns should be taken into account for *T. dimidiata* entomologic surveillance programs, as focusing surveillance efforts on houses close to street light sources may increase the sensitivity of the detection of low levels of infestation. Further studies including this pattern may also allow the elaboration of more precise risk maps at a village level [34], [35].

In conclusion, we presented here the first field evidence that street lights can directly influence house infestation by *T. dimidiata*, as the proximity of a street light significantly increased the probability of a house being infested. These field observations were confirmed in behavioral assays in a dual-choice chamber, and warrant further studies on the role of light in *T. dimidiata* dispersal. These results have important implications for the risk of *T. cruzi* transmission to households, and suggest that light spatial patterns should be taken into account by vector control programs. Indeed, this information may allow a better detection of infestation, leading to improved risk maps. Further research may also provide new opportunities exploit vector dispersal behavior for an improved control of house infestation as previously suggested [46].

## Supporting Information

Video S1 Example of a video recording showing an adult *T. dimidiata* attracted to white light in the dual choice chamber.
(MP4)
